# Closely Packed Stretchable Ultrasound Array Fabricated with Surface Charge Engineering for Contactless Gesture and Materials Detection

**DOI:** 10.1002/advs.202303403

**Published:** 2024-02-13

**Authors:** Ankan Dutta, Zhenyuan Niu, Abu Musa Abdullah, Naveen Tiwari, Md Abu Sayeed Biswas, Bowen Li, Farnaz Lorestani, Yun Jing, Huanyu Cheng, Senhao Zhang

**Affiliations:** ^1^ Department of Engineering Science and Mechanics The Pennsylvania State University University Park State College PA 16802 USA; ^2^ Center for Neural Engineering The Pennsylvania State University University Park State College PA 16802 USA; ^3^ Center for Research in Biological Chemistry and Molecular Materials (CiQUS) University of Santiago de Compostela Santiago de Compostela 15705 Spain; ^4^ Graduate Program in Acoustics The Pennsylvania State University University Park State College PA 16802 USA; ^5^ Suzhou Institute of Biomedical Engineering and Technology University of Science and Technology of China School of Biomedical Engineering 165085, 88 Keling Rd, Huqiu District Suzhou Jiangsu 215163 China

**Keywords:** closely packed ultrasound array, gesture recognition and material detection, non‐contact triboelectricity, surface charge engineering

## Abstract

Communication with hand gestures plays a significant role in human‐computer interaction by providing an intuitive and natural way for humans to communicate with machines. Ultrasound‐based devices have shown promising results in contactless hand gesture recognition without requiring physical contact. However, it is challenging to fabricate a densely packed wearable ultrasound array. Here, a stretchable ultrasound array is demonstrated with closely packed transducer elements fabricated using surface charge engineering between pre‐charged 1–3 Lead Zirconate Titanate (PZT) composite and thin polyimide film without using a microscope. The array exhibits excellent ultrasound properties with a wide bandwidth (≈57.1%) and high electromechanical coefficient (≈0.75). The ultrasound array can decipher gestures up to 10 cm in distance by using a contactless triboelectric module and identify materials from the time constant of the exponentially decaying impedance based on their triboelectric properties by utilizing the electrostatic induction phase. The newly proposed metric of the areal‐time constant is material‐specific and decreases monotonically from a highly positive human body (1.13 m^2^ s) to negatively charged polydimethylsiloxane (PDMS) (0.02 m^2^ s) in the triboelectric series. The capability of the closely packed ultrasound array to detect material along with hand gesture interpretation provides an additional dimension in the next‐generation human‐robot interaction.

## Graphical abstract

A stretchable ultrasound array, created through surface charge engineering, demonstrates effective contactless hand gesture recognition and material detection. The array demonstrates excellent ultrasound properties, interprets gestures up to 10 cm away, and identifies materials based on their triboelectric properties. The proposed material‐specific areal‐time constant metric enhances human‐robot interaction, offering a new dimension in communication.

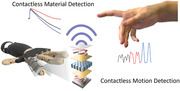

## 1 Introduction

The rapid growth and commercialization of the immersive augmented and virtual reality (AR/VR) industry (with a cumulative annual growth rate of 25.3% to a reach market size of $114.5B by 2027)^[^ ^1^ ^]^ demands improvement in the natural and intuitive communications between humans and machines. The human‐robot interaction (HRI) has also gained significant attention in recent years due to its potential applications in various fields, including gaming,^[^ ^2^, ^3^, ^4^ ^]^ healthcare,^[^ ^5^, ^6^, ^7^, ^8^, ^9^, ^10^, ^11^, ^12^ ^]^ and emergency rescues.^[^ ^3^, ^13^, ^14^, ^15^ ^]^ As a primary mode to understand and mimic human‐human interaction since the beginning of human cognition, hand gesture recognition (HGR) has been the holy grail of HRI because of its efficient communication in rugged environments that are difficult with verbal or facial expressions (e.g., construction sites and emergency rescues).^[^ ^3^, ^16^, ^17^, ^18^, ^19^, ^20^, ^21^, ^22^, ^23^ ^]^ Conventional methods for HGR typically rely on the use of visual or infrared cameras^[^ ^24^ ^]^ electromyography (EMG) measurements,^[^ ^24^, ^25^ ^]^ or stretchable strain sensors^[^ ^26^ ^]^ with resources‐intensive machine learning algorithms to decipher the gestures. However, the accuracy of HGR has been hindered because of the limited visual image quality due to environmental interference, poor contact impedance, and low‐quality data due to cross‐talks in EMG and strain sensors.^[^ ^2^, ^3^, ^24^, ^27^ ^]^

Recently, ultrasound‐based techniques traditionally used for medical imaging^[^ ^28^, ^29^, ^30^, ^31^ ^]^ have emerged as a promising alternative due to their ability to capture hand movements in 3D without physical contact and robustness to lighting conditions and occlusions.^[^ ^4^, ^32^, ^33^, ^34^, ^35^ ^]^ Ultrasound‐based HGR involves the use of high‐frequency sound waves to create a 3D image of the hand and track its movements. For instance, Chirp,^[^ ^36^ ^]^ a startup company, has leveraged micro‐electromechanical systems (MEMS) and the time‐of‐flight ultrasound sensor for touch‐free HGR. Recently, IBM^[^ ^23^ ^]^ has developed ultrasound‐based microphones to detect finger bone‐conducted sound generation during hand gestures. The ultrasound array has also shown high recognition accuracy in deciphering distinctive gestures and types of digit flexion using traditional imaging modalities. Furthermore, a wearable ultrasound array can analyze the surface geometry of the hand during gesture by using acoustic beamforming to steer and focus on the region of interest.^[^ ^16^ ^]^ Recently, contact‐free HGR using the frequency‐hopping mechanism can also alleviate frequency fading, avoiding signal interference.^[^ ^37^ ^]^ However, the ultrasound array requires the pitch size to be comparable to or less than the wavelength in the acoustic medium to avoid grating lobes that result in a low signal‐to‐noise ratio (SNR) and poor image quality.^[^ ^29^, ^31^ ^]^ Therefore, it is highly desirable to fabricate a densely packed wearable ultrasound array with the close placement of transducer elements. Moreover, high‐frequency ultrasound (>1 MHz) is essential to achieve low impedance at its resonant frequency, which requires a pitch of ∽150–200 µm generally performed by human hand placement. Although dicing saws have been exploited for transducer placement, customized saw thickness is required to tailor the pitch or the operational frequency, increasing both startup and recursive costs to create challenges in future commercialization. Therefore, there is an urgent need for an alternative and effective method to fabricate a densely packed ultrasound array. Moreover, performing HGR using imaging modalities demands sophisticated algorithms and expensive and bulky data acquisition systems, which may not be practical during daily operations.

A significant limitation of current immersive HRI technology is its inability to deliver information regarding interacting materials, limiting it to only visual information. Material detection can add an extra communication channel between humans and machines, which will also aid the Artificial Intelligence (AI) algorithm^[^ ^38^ ^]^ to better understand interaction dynamics given the material's nature. Conventional image processing and computer vision algorithms rely on the lighting conditions, orientations, colors, and textures of items based on their respective training datasets. However, it becomes challenging to categorize items having similar texture and color but with different material properties. For example, glass and clear acrylic would be indistinguishable in conventional image processing classifications, thereby AI‐controllers would misinterpret the object's weight and the nature of its interaction. Yet, incorporating an additional device for quantitative characterization of the material increases the complexity of the system. Furthermore, most advanced material characterization techniques are not feasible in practical implementation as HRI applications require contactless interaction. Therefore, a low‐cost, easily deployable, multifunctional device that can perform the contactless gesture and material detection becomes extremely important. Recently, the paper‐based non‐contact triboelectric sensing (NCTS) technology has been developed for human motion tracking^[^ ^39^ ^]^ and to decipher the walking gait cycle, direction, and speed using voltage signals.^[^ ^40^ ^]^ Using multimodal, flexible tactile, and touchless HGR with the NCTS technology also provides contactless human‐in‐the‐loop locomotion for the soft robot manipulator.^[^ ^41^ ^]^ On the other hand, non‐contact triboelectricity has also been explored in non‐contact energy harvesting systems.^[^ ^42^, ^43^ ^]^ These systems offer high flexibility and long life, and promote green energy to reduce dependence on non‐renewable resources. However, the electrostatically induced touchless gesture sensor^[^ ^44^, ^45^, ^46^ ^]^ has not fully exploited the charge transfer dynamics in the stationary phase that unveils important material properties such as electro‐affinity or work function due to triboelectricity.^[^ ^44^, ^47^, ^48^, ^49^, ^50^ ^]^ The time constant of the exponentially decaying electrostatically induced charge depends on the material's work function, thus enabling the possibility of detecting the material along with the motion or gesture.

**Figure 1:**
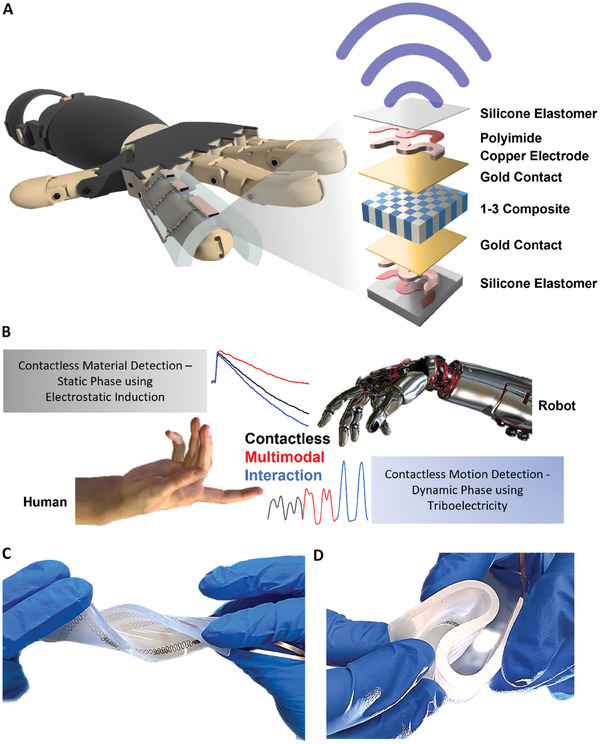
Design and working principle of the stretchable ultrasound array with closely packed transducer elements for the non‐contact gesture and material detection. A) Schematic of the stretchable ultrasound array consisting of silicone elastomer, 1–3 PZT composite with gold contacts, copper/polyimide (Cu/PI) electrode, and thin silicone elastomer, placed on the index finger of a robot. B) The sensor on the index finger of a robot recognizes gestures and materials based on triboelectricity and electrostatic induction to communicate with a human, facilitating human‐computer interaction. The ultrasound array with transducer elements interconnected by Cu/PI electrodes can undergo C) twisting and D) multiple bending without a fracture.

Herein, this work demonstrates a stretchable ultrasound array with closely packed transducer elements fabricated by leveraging the surface charge engineering between the 1‐3 lead zirconate titanate (PZT) composite and thin polyimide (PI) film without using a microscope. With a tunable gap between two adjacent transducer elements or kerf down to 20 µm, the array using PZT with a thickness of 420 (or 200) µm shows a central frequency of 3.94 (or 7.3) MHz with an excellent bandwidth of ≈57.1% (or 52.3%) and high electromechanical coefficient of ≈0.75 (or ≈0.56). The resulting multimodal flexible and stretchable ultrasound array could decipher material characteristics and hand gestures based on non‐contact triboelectrification for HRI applications (**Figure** **1**). Different orientations and gestures such as punch, waving hand, sliding left and right, spread, and pinch could be interpreted at a distance up to 7 cm. Along with gesture recognition, the stretchable array could also estimate the triboelectricity of the material by evaluating the time constant of exponentially decaying impedance during the electrostatic induction phase, independent of force or pressure applied. The areal‐time constant (area multiplied by time constant) is material‐specific and decreases monotonically from a highly positive human body (1.13 m^2^ s) to negatively charged polydimethylsiloxane (PDMS) (0.02 m^2^ s) in the triboelectric series. The multimodal decoupling^[^ ^51^ ^]^ with contactless ultrasound array in triboelectric material characteristics and gesture recognition could pave the way for next‐generation HCI.

## 2 Experimental Section

### 2.1 Materials

Prime‐graded silicon (Si) substrate (p‐doped, 100) was purchased from Alpha Nanotech, USA. PDMS kit (SYLGARD 184 Silicone Elastomer) was purchased from the Dow Chemical Company, USA. The piezoelectric 1–3 PZT‐5A composite transducers with a thickness of 200 and 420 µm were purchased from Del Piezo Specialties, USA. The transducers were diced using an ADT 7100 dicing saw (thickness 150 µm), Advanced Dicing Technologies, Israel. Ecoflex 0030 (Part A and B) was purchased from Smooth‐On, USA. The Pyralux copper/polyimide film (Cu/PI of 9 µm/12 µm) and Kapton PI film (thickness: 12, 30, 50, and 100 µm) were purchased from DuPont, USA. The water‐soluble tape 5414 was obtained from 3M, USA. The silver epoxy EPO‐TEK H20E was purchased from Epoxy Technologies, USA. The isopropyl alcohol (IPA) was purchased from VWR International, USA.

### 2.2 Fabrication

#### 2.2.1 Fabrication of Closely Packed Ultrasound Transducer Elements

**Figure 2:**
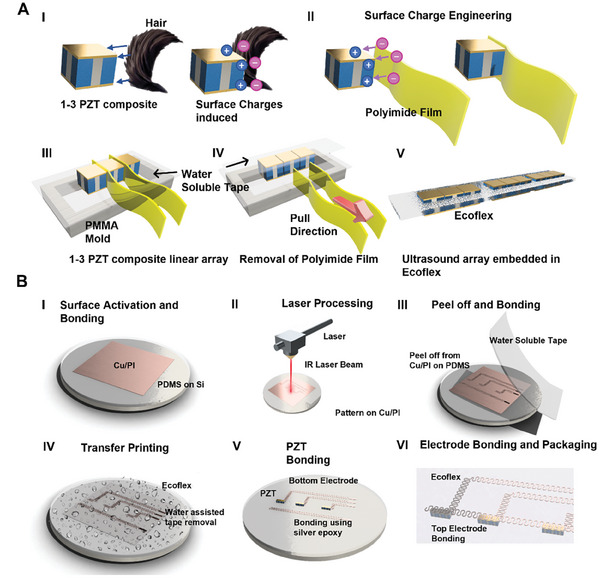
Fabrication procedure of a stretchable ultrasound array with closely packed transducer elements. A) After I) pre‐charging the 1–3 PZT composite with hair, II) surface charge engineering allows the attachment of PI film with PZT to form III) PZT/PI/PZT array. IV) Removing the PI and embedding the PZT array in a silicone elastomer forms a closely packed ultrasound array. B) The stretchable device fabrication starts with I) laminating and II) laser scribing the Cu/PI film placed on a stretchable elastomeric substrate to form the patterned top or bottom electrode. After III) transfer printing the patterned Cu/PI with water‐soluble tape and IV) dissolving the tape, V) the previously prepared ultrasound array with closely packed transducer elements was bonded to the bottom/top electrodes using silver paste and irrigated with Ecoflex to form VI) a stretchable ultrasound array.

The diced 1–3 PZT composite was rubbed with hair samples to engineer surface charges on its side surfaces (**Figure** **2**A‐I). The positively charged 1–3 PZT composite was then placed sufficiently close to a negatively charged PI thin film (thickness: 12 or 30 µm) for attachment due to normal electrostatic force (Figure 2A‐II). Repeating the steps aligned and attached another 1–3 PZT composite to the thin PI, forming a closely packed PZT/PI/PZT. Next, a thin polymethyl methacrylate (PMMA) mold was prepared with a hollow opening covered by water‐soluble tape on which the aligning experiment was performed. Similarly, a water‐soluble tape was used again as a mask for the top gold contact along with the previously placed water‐soluble tape, thus protecting both the top and bottom contacts (Figure 2A‐III). After raising the temperature to 100 °C, the PI film was easily removed from the PZT/PI/PZT to form closely packed transducer elements. For PZT with a thickness of 200 µm, the water‐soluble mask was placed on the top gold contact after the PI removal, whereas the 420 µm‐thick PZT used the water‐soluble mask on top contact before the PI removal (Figure 2A‐IV). Then, the Ecoflex precursor was poured into the mask‐protected 1–3 PZT composite array and suspended using water‐soluble tape on the PMMA mold. After curing at 80 °C for 15 min, the sandwiched 1–3 PZT composite array was kept in water to dissolve the water‐soluble mask. Removing the PMMA mold resulted in a flexible array with closely packed 1–3 PZT composite elements embedded in the cured Ecoflex matrix (Figure 2A‐V; Figure S1, Supporting Information). The whole fabrication process was performed without using any microscope.

#### 2.2.2 Fabrication of the Stretchable Ultrasound Arrays with Closely Packed Transducer Elements

The fabrication started by spin‐coating (SPIN150i, RK‐AHT, Germany) the PDMS precursor solution (10:1) at 800 rpm for 30 s on a Si substrate. The Cu/PI film was surface activated using ultraviolet light (PSD Series Digital Ultraviolet Ozone System, Novascan, USA) for 3 min for bonding with PDMS/Si (Figure 2B‐I). The bonded Cu/PI on the PDMS/Si substrate was pulse ablated (SPI Lasers, UK) to engrave the top or bottom electrode (Figure 2B‐II). The laser parameters (wavelength: 1040 – 1200 nm, pulse energy: 0.4 mJ, pulse duration: 250 ns, speed: 200 mm ^−1^s) were optimized to maximize the yield of ablated Cu similar to those reported previously.^[^ ^52^, ^53^, ^54^ ^]^ After laser ablation of Cu/PI, the unwanted carbon formed on the PI side was removed using IPA. A thicker Ecoflex film (1:1) was prepared on Si substrate at 1000 rpm for 30 s for the bottom electrode, whereas a thinner Ecoflex film (1:1) was spin‐coated on PMMA substrate (for improved transparency during placement) at 2000 rpm at 60 s for the top electrode. The patterned top and bottom Cu/PI electrodes were transfer‐printed from donor PDMS to receiver Ecoflex substrate using water‐soluble tape after 7 min of surface activation (Figure 2B‐III). After dissolving water‐soluble tape with water (Figure 2B‐IV), the previously prepared 1–3 PZT composite transducer array (thickness of 200 or 420 µm) was aligned and bonded to the bottom electrode using the silver epoxy EPO‐TEK H20E followed by baking at 150 °C for 10 min (Figure 2B‐V). Similarly, the top electrode on the transparent Ecoflex/PMMA substrate was aligned with the 1–3 PZT composite array and bonded using silver epoxy. Next, the device between the top PMMA and bottom Si substrates was uniformly packaged using Ecoflex without forming any bubbles. After curing Ecoflex at 80 °C for 15 min, both substrates were separated to result in a stretchable ultrasound array (Figure 2B‐VI; Figure S2, Supporting Information).

### 2.3 Characterization

#### 2.3.1 Optical Characterization of Packaging of Ultrasound Arrays

**Figure 3:**
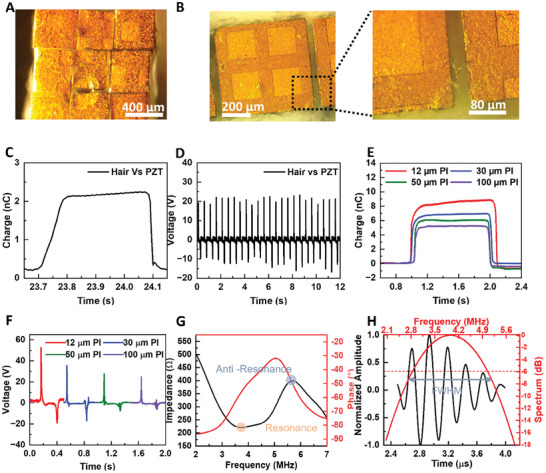
Optical, triboelectric, and ultrasound characterizations of the stretchable ultrasound array. The optical images of a closely packed ultrasound array using A) 12 µm PI and (B) 30 µm PI for surface charge engineering. Triboelectric characterization of the human hair sample and PZT, showing C) charge retention up to 2 nC and D) peak output voltage ≈20 V. Triboelectric characterization of PI with different thicknesses relative to PZT, indicating the decreased E) charge retention and F) peak voltage as the PI thickness increases from 12 to 100 µm. G) Impedance and phase angle spectrum of 1–3 PZT composite with a thickness of 420 µm (resonant and anti‐resonant frequencies marked with shaded circles). H) Time and frequency domain characterizations show an excellent bandwidth of 57.1% and a spatial time pulse of 1.5 µs.

Because of manual operation in the low‐cost fabrication processes and the nonvanishing gap between the PZT and PI thin film (thickness of 12 or 30 µm) in the closely packed ultrasound array (thickness of 420 µm), the gap between two adjacent transducer elements or kerf is larger than the thickness of the PI film. The optical images obtained from Digital Microscope (AmScope, USA) showed a kerf of ≈20 (or 40) µm between the 12 (or 30) µm‐thick PI film and PZT in the PZT/PI/PZT stack array (**Figure** **3**A,B) due to the higher (or lower) triboelectric effect.

#### 2.3.2 Triboelectric Characterization of the PZT and Hair

The surface charge engineering or electrification of PZT using hair based on the difference between their triboelectric properties^[^ ^47^, ^55^, ^56^, ^57^ ^]^ was characterized by using a triboelectric nanogenerator (TENG). PZT and hair stacks were attached to Cu electrodes for constructing the TENG (Figure S3, Supporting Information). The TENG was pressed and released at an interval of 0.5 s to observe the charge transfer. The TENG was further tapped at a frequency of 2 Hz for voltage measurement (an air gap of 0.5 cm between the two films). Because PZT is triboelectrically positive compared to hair,^[^ ^47^, ^58^ ^]^ positive charges are induced on the active boundary of the PZT transducer, with negative charges on the active region of the hair. The contact electrification between hair and PZT gives a surface charge transfer density of 29 µC m^−2^ measured using Autolab (Metrohm) (Figure 3C) and an open‐circuit voltage of ≈20 V measured using an oscilloscope (SDS1202X‐E, Siglent Technologies) (Figure 3D).

#### 2.3.3 Triboelectric Characterization of PI and PZT

Close placement of the positively charged PZT transducer elements near the negatively charged PI thin film^[^ ^58^, ^59^, ^60^, ^61^, ^62^ ^]^ produced the electrostatic attraction force that depends on the charge transfer between the active surface of two materials. The surface charge transfer density between PI film and PZT decreases from 115.3 to 68 µC m^−2^ as the thickness of the PI film increases from 12 to 100 µm measured using Autolab (Metrohm) (Figure 3E). The open‐circuit voltage also drops from 53 to 25 V due to the increased thickness from 12 to 100 µm (Figure 3F). Therefore, the electrostatic force is sufficient to hold a thin PI film of 12 µm against its gravity (Figure S4, Supporting Information) but fails to attach the PI film of 100 µm (Figure S5, Supporting Information).

#### 2.3.4 Ultrasound Characterization of Closely Packed Ultrasound Arrays

The fabrication of a stretchable, closely packed high‐frequency ultrasound array facilitated by surface charge engineering is frequency‐adaptable with consistent acoustic performance for both thick (420 µm) and thin (200 µm) 1–3 PZT composite arrays. The thick 1–3 PZT composite showed a resonant frequency of 3.7 MHz and an anti‐resonant frequency of 5.63 MHz, resulting in an exceptionally high effective electromechanical coefficient of 0.75 (Figure 3G). The impedance and phase angle spectra were characterized using an LCR Meter (IM 3536‐01, Hioki). In contrast, the thin 1–3 PZT composite results in a higher resonant frequency of 7.4 MHz and an anti‐resonant frequency of 9 MHz, with an effective electromechanical coefficient of 0.56 (Figure S6, Supporting Information). The pulse‐echo response of the thick 1–3 PZT composite corresponds to a central frequency of 3.94 MHz with an excellent wide bandwidth of 57.1% and a spatial pulse length of 1.5 µs characterized using pulser‐receiver (Panametric 5077PR, Olympus) (Figure 3H). In comparison, the pulse‐echo response of the thin 1–3 PZT composite shows a central frequency of 7.3 MHz with a wide bandwidth of 52.3% and an extremely narrow spatial pulse length of 0.5 µs (Figure S7, Supporting Information). The excellent ultrasound characteristics of the fabricated closely packed ultrasound array are consistent with recent literature reports.^[^ ^6^, ^29^, ^63^ ^]^

#### 2.3.5 Contactless Triboelectric Characterization of Closely Packed Ultrasound Arrays

Acting as a non‐contact triboelectric nanogenerator,^[^ ^64^, ^65^ ^]^ the stretchable ultrasound array embedded in the silicone elastomer provides opportunities for contactless gesture and material recognition. The dynamic gesture contributes to the triboelectrification of the device, resulting in changes in capacitance, thus, the measured impedance using an LCR Meter (IM 3536‐01, Hioki). The impedance analysis of the ultrasound array was performed by providing 1 V across the electrode with corresponding resonant frequencies, i.e., 7.3 MHz for 200 µm and 3.9 MHz for 420 µm 1–3 PZT composite array, respectively.

## 3 Results and Discussion

### 3.1 Working Mechanism of Contactless Triboelectrification

**Figure 4:**
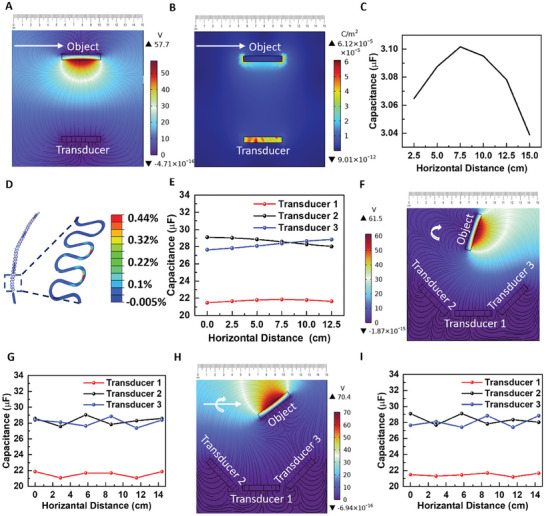
The working mechanism of gesture recognition. A) Potential distribution and B) displacement current norm obtained from COMSOL simulation for contactless triboelectrification between the object and transducer with C) capacitance dependent on the distance between the object and transducer. D) The maximum principal strain distribution in the Cu interconnects during bending. E) The slope in the simulated capacitance versus horizontal distance curves from an array of three transducers implies the direction of the object is from transducer 2 to 3. F) The clockwise rotation of the object can be mapped by comparing G) the phase shift in the symmetrical capacitance oscillation between transducers 2 and 3. The simulated H) potential distribution and I) derived capacitance to decipher mixed linear and rotational motions.

To avoid sophisticated imaging algorithms and a data acquisition system in imaging‐based gesture recognition, a simple, contactless, stretchable gesture recognition sensor based on the closely packed ultrasound array with a consistent and excellent acoustic property is demonstrated. The recognition is based on coupled triboelectrification and electrostatic induction, which depends on the projected area and the distance between the transducer and the object.^[^ ^44^, ^64^, ^65^, ^66^ ^]^ Because the frequency range of the ultrasound (7.3 MHz) is orders of magnitude higher than the frequency of the human gesture (≈1 Hz), the impedance variation with a gesture cannot be realized using a time‐of‐flight mechanism as revealed by the simulation in COMSOL (Figure S8, Supporting Information). To simulate contactless triboelectricity, both the object and the transducer are grounded. A potential of 1 V is applied across the transducer, and a surface charge density of 25 µC m^−2^ is applied to the object (experimentally validated by the charge transfer from the human hand to the ultrasound array) (Figure S9, Supporting Information). The simulated capacitance decreases with the increasing distance between the object and the ultrasound array (Figure S10, Supporting Information) and increases with the increasing area of the object (Figure S11, Supporting Information). The simulated potential distribution (**Figure** **4**A) and normalized displacement current norm (Figure 4B) for contactless triboelectrification between the object and transducer generate the capacitance variation due to the horizontal motion of the object. The capacitance reaches the maximum when the object is closest (horizontal distance of 7.5 cm from the left boundary) to the transducer (Figure 4C), which is consistent with the mechanism of electrostatic induction or capacitive sensing (capacitance is inversely proportional to distance).

### 3.2 Mechanical Simulation of Stretchable Ultrasound Arrays

Compared with a single transducer, an array of transducers can provide more precise information regarding the object's projected area and distance for capacitive sensing. The design of the array needs to first consider the mechanical stability of the large stretchable array.^[^ ^3^, ^18^, ^67^ ^]^ In the proof‐of‐the‐concept demonstration, the closely packed ultrasound array is designed to be placed on the three phalanges – distal, middle, and proximal – of the index finger of an adult male. The serpentine interconnects connect the three closely packed ultrasound arrays, increasing the ultrasound array's mechanical stretchability. The mechanical simulation of the device (cross‐section shown in Figure S12, Supporting Information) using Abaqus shows that the maximum principal strain in Cu/PI interconnects (Figure S13, Supporting Information) upon finger bending with a radius of 3.5 cm and 2.5 mm locally at the finger joint (3 and 5 cm from the Meta Carpo Phalangeal Joint, making 30° relative to each other, as shown in Figure S14, Supporting Information) is only 0.44%, which is much lower than its fracture strain (Figure 4D).

### 3.3 Working Mechanism of Gesture Recognition Using Ultrasound Arrays

After placing the stretchable ultrasound array on the index finger with different phalanges orienting at different angles, the projected area and distances between the device and the object are changed. The precise location and direction of the object's motion can be analyzed by simultaneously sampling the impedance and capacitance of the ultrasound array. For example, three transducers placed at different orientations (Figure S15, Supporting Information) can help decipher the object's horizontal motion. The smaller distance between the object and transducers 2 and 3 results in higher capacitance than that for transducer 1. Moreover, the increasing capacitance for transducer 3 and decreasing capacitance for transducer 3 with almost unchanged capacitance for transducer 1 signifies that the object's moving direction is from transducer 2 to 3 (Figure 4E). Along with horizontal movement, the capacitances of the three transducers can also interpret the direction of rotation (Figure 4F). The clockwise rotation of the object can be mapped by comparing the phase shift in the symmetrical capacitance oscillation between transducers 2 and 3, where transducer 2 reaches its maximum value (at 6 cm) before transducer 3 (at 9 cm) (Figure 4G). As a result, the array can be used to decipher any general motions, which can be represented as a combination of linear and rotational motions (Figure 4H). For instance, the initial (at 0 cm) higher value of transducer 2 together with reaching its maximum value (at 6 cm) before transducer 3 (at 9 cm) infers that the object moves to the right with clockwise rotation (Figure 4I). The simulation with just three transducers shows that an ultrasound array can be used to interpret the general motion of the object, even without any imaging technique.

### 3.4 Output Performance of Contactless Gesture Recognition

**Figure 5:**
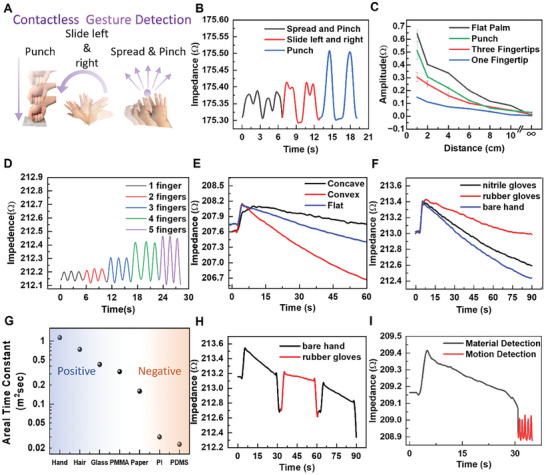
The output performance of the stretchable ultrasound array to recognize gestures and decipher the material. A) The stretchable array could interpret gestures of punch, slide left and right, and spread & pinch, with B) different waveforms depending on the distance, projected area, and dynamics. C) The vertical movement of gestures with the flat hand, fist, three, and one fingertip shows decreased impedance amplitude with distance. D) The impedance amplitude increases with the number of fingers during horizontal finger movement. E) The time decay constant from an exponentially decaying impedance determines the hand's orientation: concave, convex, and flat. F) The decay signature of impedance in the exponential pattern depends on the material: bare hand versus nitrile and rubber gloves. G) The areal‐time decay constant decreases monotonically according to the triboelectric series and H) its use for dynamic detection of the change of material. I) The use of the areal‐time constant and peak voltage from a static‐dynamic sequence to simultaneously determine the material and gesture at a distance of 10 cm from the ultrasound array.

The proof‐of‐the‐concept demonstration for gesture recognition is highlighted by a few commonly used gestures such as vertical punch, slide right‐left, and spread‐pinch.^[^ ^16^, ^17^, ^37^, ^68^ ^]^ The temporal dynamics of these gestures (**Figure** **5**A) can be observed by the measured impedance. Different from spread‐pinch and slide left‐right gestures that are presented at ≈4 cm above the sensor, the vertical punch gesture varies in distance to result in an increased amplitude of ≈0.2 Ω (Figure 5B). The vertical movement of gestures with the flat hand, punch, three, and one fingertip at 2 Hz exhibits reduced impedance amplitude due to decreased projected area, which all diminishes with the increase in the distance (Figure 5C and Figure S16, Supporting Information). For example, a flat hand covers ≈95 cm^2^ to result in an impedance amplitude of 0.65 Ω, whereas one fingertip, comprising ≈3.5 cm^2^ surface area, produces an amplitude of 0.15 Ω at 1 cm above the sensor. The contactless gesture recognition sensor works for a distance of ≈10 cm in length above the sensor. For the horizontal movement of fingers, the impedance amplitude increases from 0.07 to 0.36 Ω as the number of fingers (placed 4 cm above at a frequency of 0.5 Hz) increases from one to five (Figure 5D), which is consistent with the tread observed in Figure 5C.

Although the triboelectrification between the transducer and the object depends on the projected area and distance, it cannot determine the hand's orientation or curvature (negative vs positive) (Figure S17, Supporting Information). To address this challenge, we exploit the time decay constant from an exponentially declining impedance or exponentially increasing capacitance.^[^ ^44^, ^49^, ^55^ ^]^ By keeping the object at rest (or stationary) with respect to the transducer, electrostatically induced charges at the transducer surface would result in different time constants for estimating the curvature of the hand's orientation. The time decay constant τ in the exponentially decaying impedance *Z*  = *Z*_0_ *e* ^−*t*/τ^ increases from 72.7 to 366.3 s as the hand switches from the convex (fitting exponential data with R^2^ = 0.9998) to concave orientation (R^2^ = 0.991) (Figure 5E). The differences in decay profiles (or the dynamics of exponential charge decay) are attributed to the different electrostatic field distributions modulated by the curvature of the hand (Note S1 and Figure S18, Supporting Information).

### 3.5 Output Performance of Contactless Material Characterization

Keeping the object stationary to the sensor provides the exponential decay of impedance, whereas the triboelectrification induced by the dynamic motion of the object leads to local oscillation of the impedance, with the mean impedance remaining constant globally. The exponential decay signature of impedance also depends on the material (e.g., nitrile and rubber gloves vs bare hand) and the trend of decay is consistent with the triboelectric series^[^ ^47^, ^58^ ^]^ (Note S2, Supporting Information). The highly positive bare human hand has a decay constant of 104.8 s, whereas the negatively charged rubber shows a decay constant of 86.9 s (Figure 5F). Although prior contamination with other triboelectrically charged materials changes the initial impedance (or surface charge density), the time decay constant remains unchanged (Note S3 and Figure S19, Supporting Information). The decay in complex impedance *Z* of the ultrasound array signifies an increase in the capacitance *C* of the ultrasound element across the top and bottom electrodes. The result implies the equivalent capacitor between the device and the human finger is charging the ultrasound capacitor while discharging itself following the equation:

(1) $$Z\;=\;R-\frac{j}{\omega C}+j\omega L$$

where ω is the operational frequency of the device and *L* is the device inductance that is considered as a constant. As the voltage supplied across the electrodes is constant (1 V), the decrease in capacitance accounts for the charge transferred. Therefore, the exponential decay in the impedance is due to the exponential decay in the charge from the human body surface, which is consistent with charging decay in the electron thermionic emission mode (Note S4, Supporting Information). The surface charge *Q_s_* during the thermionic emission is given by the differential equation^[^ ^44^, ^45^, ^50^, ^66^ ^]^

(2) $$\frac{1}{A_{0}AT}\;\frac{\partial Q_{s}}{\partial t}=\frac{\lambda_{1}}{k}Q_{s}e^{\frac{W}{kT}}$$

where *A*_0_ is the Richardson constant of a free electron, *T* is the temperature, *A* is the surface area, *W* is the height of the potential barrier, λ_1_ is approximately a constant given by λ_1_ =  λΔ*W*/*Q_s_*, with Δ*W* as the change in the potential barrier height due to the surface electric field and λ as the material‐specific factor, and *k* is the Boltzmann factor. Solving Equation (1) gives the surface charge *Q_s_* that decays exponentially following

(3) $$Q_{s}=e^{-ASt}\;Q_{s0}$$

where *S* is the material‐specific constant given by $S\;=\lambda_{1}\;A_{0}Te^{\frac{W}{kT}}/k$. The device‐human body capacitor charges the ultrasound capacitor and exponentially increases the surface charge (Δ*Q_s_* =  Δ*CV*, where *V*  =  1). The exponential increase in the capacitor results in the exponential decay of impedance *Z*  =  1/ω*C*. Therefore, the time decay constant τ is given by τ  =  1/*AS*, implying that the areal‐time decay constant $\tau A\;=\;1/S\propto e^{-\frac{W}{kT}}$ is a material‐specific constant rather than a simple time decay constant. By fitting the exponentially decaying impedance, the areal‐time decay constant decreases monotonically from the highly positive human body (1.13 m^2^ s) to negatively charged PDMS (0.02 m^2^ s) (Figure 5G).

### 3.6 Sequential Contactless Material and Gesture Recognition

The material and gesture recognition with the stretchable ultrasound array provides the possibility to holistically detect the object's chemical composition and physical motion sequentially. The device could dynamically detect the change of material by analyzing the variation in the impedance slope. The time constant in the exponentially decaying impedance increases from 87.4 s (R^2^ = 0.981) for rubber gloves to 105.2 s (R^2^ = 0.998) for bare hands (Figure 5H). The material and gesture can be sequentially deciphered by following the analysis sequence from the static to the dynamic phase. The static phase is utilized for material detection by extracting the time decay constant of 105.93 s (R^2^ = 0.994) and an amplitude of 0.15 Ω (Figure 5I). Given that the area of the object is 108 cm^2^, the material is confirmed to be a human hand according to the areal‐time decay constant of 1.14 m^2^ s. Moreover, the gesture is predicted to be three fingers with prior information about the object's distance of 4 cm from the device. By exploiting the temporal impedance readout from the ultrasound array, the projected area and distance of the object can be dynamically estimated. Following the estimated projected area and distance, the static‐dynamic analysis sequence can be used to retrieve the areal‐time constant and impedance waveform, deciphering the material property and relative motion or gesture. The coupled material‐motion signals from the multimodal sensor can be fed to the image processing algorithms to confirm the motion and further enable a material map to estimate different interacting materials. Typically, mobile cameras can capture the object's shape and color in RGB format and depth using the depth sensor. The imaging technology can be further enhanced by complementing electrostatic‐based mapping technology. In the dynamic phase, the multimodal sensor can decipher gestures, motion, and area, whereas, in the stationary phase, the sensor can capture the areal‐time constant across the surroundings to map the material composition of the interacted objects. However, execution of the material mapping using a single transducer is challenging at the present stage of the development. Therefore, it is imperative to further develop and deploy arrays with novel algorithms in practical scenarios.

## 4 Conclusion

In summary, a flexible and stretchable ultrasound array has been fabricated by leveraging surface charge engineering to tightly pack transducer elements based on 1–3 PZT composites with a tunable kerf down to 20 µm without a microscope. The non‐contact triboelectrification‐based multimodal ultrasound sensor could detect various movements and positions, such as punching, waving, sliding left and right, and spreading & pinching, at a distance of up to 10 cm. In addition, the ultrasound array could estimate the materials' triboelectric properties by assessing the impedance decay time constant during the electrostatic induction phase. The material‐specific areal‐time constant exhibits a monotonic decrease from the highly positive human body (1.13 m^2^ s) to negatively charged PDMS (0.02 m^2^ s) across the triboelectric series. This newly introduced metric of the areal‐time constant comes from the fundamental energy‐band diagram of the material and may play a vital role in the contactless characterization of triboelectric material, independent of the force applied. Along with contactless material and hand gesture recognition, non‐contact triboelectricity can be leveraged in other applications such as energy harvesting and position/motion sensing. The ultrasound array fabricated by simple surface charge engineering with excellent acoustic properties can also find promising applications in high‐frequency biomedical imaging, drug delivery,^[^ ^69^ ^]^ and cavitation^[^ ^70^ ^]^ Along with traditional ultrasound imaging, the non‐contact triboelectric‐based gesture and material detection can aid AI‐powered controllers for improved understanding and analyzing of the human‐robot interaction with an additional dimension of material.

## Conflict of Interest

The authors declare no conflict of interest.

## Supporting information

Supporting Information

## Data Availability

The data that support the findings of this study are available from the corresponding author upon reasonable request.
