# Supplementary material for: Closely Packed Stretchable Ultrasound Array Fabricated with Surface Charge Engineering for Contactless Gesture and Materials Detection
Source: Adv Sci (Weinh). 2024 Feb 13;11(15):2303403. doi: 10.1002/advs.202303403 (PMC11022739; doi:10.1002/advs.202303403)
Supplement: Supplementary file 1 — Supporting Information [file ADVS-11-2303403-s001.pdf]

ADVANCED SCIENCE
Open Access

Supporting Information

for *Adv. Sci.*, DOI 10.1002/advs.202303403

Closely Packed Stretchable Ultrasound Array Fabricated with Surface Charge Engineering for Contactless Gesture and Materials Detection

Ankan Dutta, Zhenyuan Niu, Abu Musa Abdullah, Naveen Tiwari, Md Abu Sayeed Biswas, Bowen Li, Farnaz Lorestani, Yun Jing, Huanyu Cheng and Senhao Zhang*

Supplementary Information

Closely Packed Stretchable Ultrasound Array Fabricated with Surface Charge Engineering for Contactless Gesture and Material Detection

Ankan Dutta[1,2], Zhenyuan Niu[1§], Abu Musa Abdullah[1§], Naveen Tiwari[1,3], Md Abu Sayeed Biswas[1], Bowen Li[1], Farnaz Lorestani[1], Yun Jing[4], Huanyu Cheng[1*,] Senhao Zhang

[1]Department of Engineering Science and Mechanics, The Pennsylvania State University, University Park, 16802, USA

[2]Center for Neural Engineering, The Pennsylvania State University, University Park, 16802, USA

[3]Center for Research in Biological Chemistry and Molecular Materials (CiQUS), University of Santiago de Compostela, Santiago de Compostela, 15705, Spain

[4]Graduate Program in Acoustics, The Pennsylvania State University, University Park, Pennsylvania 16802, USA

[*]To whom correspondence should be addressed: E-mail: huanyu.cheng@psu.edu

<u>Supplementary Note (Note S1):</u> Variation of the time constant due to changes in orientation

The fringe capacitance and fringe electric field depend on the orientation (concave or convex) along with the area of the electrodes **(Fig S18)**. The COMSOL electrostatics simulation shows that the slope of the relative change in fringe capacitance due to the relative change in geometric area is approximately 22% higher for convex orientation than concave orientation (the same magnitude of the curvature for both orientations). Therefore, the effective area projected by the electrostatic field lines for convex orientation is approximately 22% higher than the concave orientation for the same geometric area ($\Delta C \propto \Delta A$). Here, the material-specific areal-time constant S depends on the material and remains the same, so the time constant τ lowers for convex orientation as $\tau A = 1/S$ remains a constant. This is consistent with the experimental result that the time decay constant increases as the hand switches from the convex to concave orientation **(Fig. 5E)**. It is important to note that only the polarity is changed to result in a change in orientation (convex and concave) in the simulation but the magnitude of curvature is kept the same. The changes in the magnitude of curvature would change the distance, dielectric material, and the slope of relative change in fringe capacitance due to relative change in the geometric area.

<u>Supplementary Note (Note S2):</u> Non-contact Triboelectric Sensor

Triboelectricity can be generated by utilizing the surface charge of materials. It can be produced through both contact and non-contact electrification. Contact triboelectricity involves direct contact and separation of two different materials (according to triboelectric series), leading to electron transfer and energy generation. Devices utilizing contact triboelectricity, such as triboelectric generators, come in physical contact to produce electric charges. On the other hand, non-contact triboelectricity operates without direct physical contact, relying on electrostatically induced surface charges from external stimuli such as vibrations or movements. Compared to contact triboelectric systems, the non-contact method offers higher durability and flexibility in applications such as touchless interfaces and energy harvesting from ambient sources. While contact triboelectricity may require maintenance due to wear, non-contact triboelectric systems are often considered more environmentally friendly due to high durability and energy harvesting from ambient movements. Capacitive sensors operate on the principle of detecting changes in capacitance, thereby changing the impedance as well. Capacitive sensors are widely used in touchscreens, proximity detection systems, and various applications where non-contact sensing is essential. Triboelectric and capacitive sensors fundamentally follow Maxwell's Laws, so it is more about the current terminology used in the literature that differentiates them, especially non-contact triboelectric and capacitive sensors. Here, charge decay in the static phase of the sensor is one of its essential aspects. Characterization of surface charge decay is not of utmost importance and, therefore, not well discussed in the capacitance sensor terminology, whereas in triboelectricity, there are several literature reports discussing increasing or decreasing charge decay[1].

<u>Supplementary Note (Note S3):</u> Invariance of the time decay constant during random surface contamination.

The material sensing mechanism relies on the dynamics of the charge decay process rather than the initial charge density. The initial charge density on the surface depends on the interacted material before the testing or experiment. The initial surface charge density can be changed by prior interacted material, object area, and distance between object and sensor. Therefore, the initial charge density is a result of the surface phenomenon, presenting challenges to determine the material in practical settings. On the other hand, the dynamics of charge decay is a bulk material characteristic independent of interacting material. The charge decay process is fitted with an exponential decay satisfying thermionic emission process2 to calculate the time constant that corresponds to the inverse of the product of object area and material-specific parameter S. Both the time constant and the material-specific parameter are independent of contacted materials **(Fig. S19)**. In this experiment, human hands with nitrile gloves contact objects such as glass, PMMA, paper, polyimide, and PDMS sequentially (triboelectrically positive to negative) for 10 seconds. After that, the hand wearing the nitrile gloves was made to hover around contactlessly over the sensor for 90 seconds. The experiment was performed sequentially, where different materials were brought into contact within 100-second intervals (10 seconds of contact and 90 seconds of contactless sensing). The exponential decay of the impedance was cumulatively fitted to different instances of contactless material detection. The exponential decay constant is approximately 98 seconds ($R^2 = 0.94$). During the intermediate contamination process, the initial surface charge or impedance is modulated and varied randomly on several instances as it depends on surface contamination and the distance between the object and the sensor (from human and experimental errors). In contrast, the exponential decay of the impedance is consistent in an average or larger time window even though there are intermediate contamination events of 10 seconds.

<u>Supplementary Note (Note S4):</u> Triboelectric charge decay in electron thermionic emission mode.

Charge decay in electron thermionic emission mode is a phenomenon characterized by the gradual dissipation of accumulated charges on a material's surface over time. In the context of thermionic emission where electrons are emitted from a material due to thermal excitation, it is imperative to understand charge decay for applications reliant on controlled electron emission. Recombination processes, involving the annihilation of electron-hole pairs, are pivotal contributors to charge decay, contingent upon intrinsic material properties and environmental parameters. The kinetics are further influenced by carrier mobility within the material. Temperature variations, exerting an influence on thermal excitation probabilities, directly modulate the rate of thermionic emission and, consequently, the associated charge decay. Non-contact triboelectric charge decay in electron thermionic emission mode involves the dissipation of accumulated charges on material surfaces without physical contact. Initially generated through processes like contact electrification, these charges undergo decay via electron thermionic emission, where thermal energy enables electrons to overcome potential barriers and escape into the environment. The rate of decay is temperature-dependent, with higher temperatures promoting faster charge dissipation. Material properties, including work function and electron mobility, significantly affect the electron emission process. Additionally, the potential barrier on the material's surface, a key determinant of electron escape, may be regulated by environmental factors such as temperature, applied electric potential.

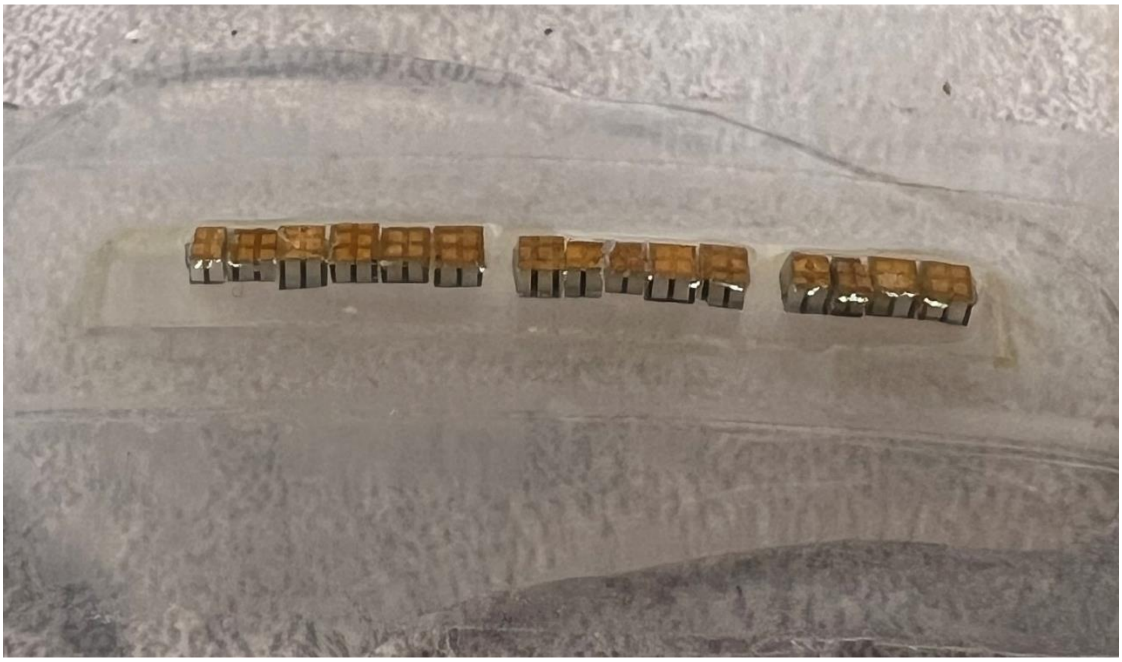

Fig S1: Flexible 1-3 PZT composite array embedded in the silicone elastomeric matrix.

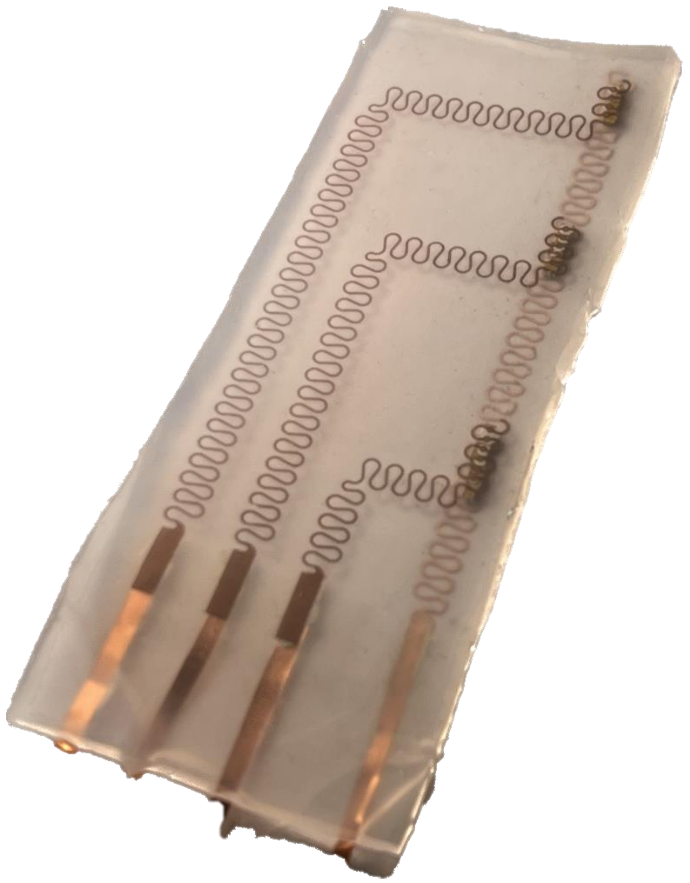

Fig S2: Optical image of the stretchable ultrasound array with closely packed transducer elements.

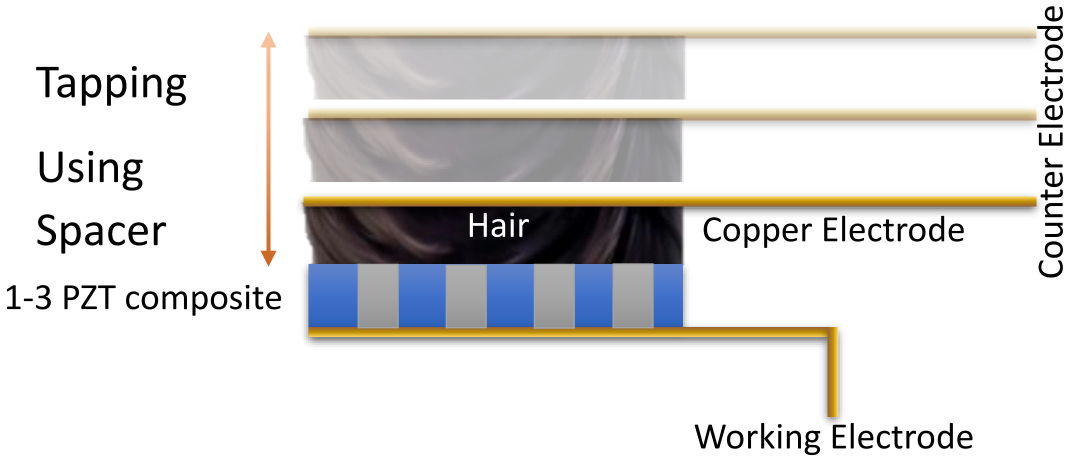

Fig S3: Schematic showing the TENG measurement setup (with Autolab) for surface electrification between 1-3 PZT composite and hair.

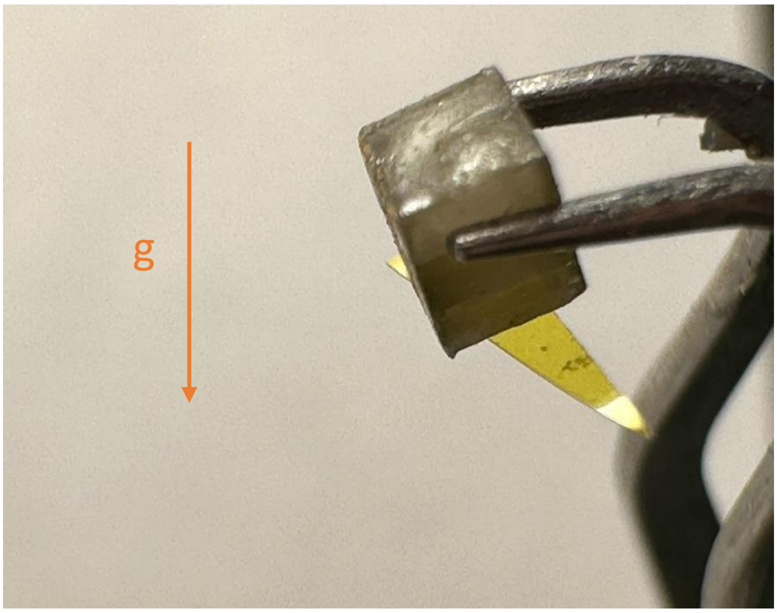

Fig S4: Image of the PZT element holding the 12 μm-think PI film against gravity with the electrostatic force.

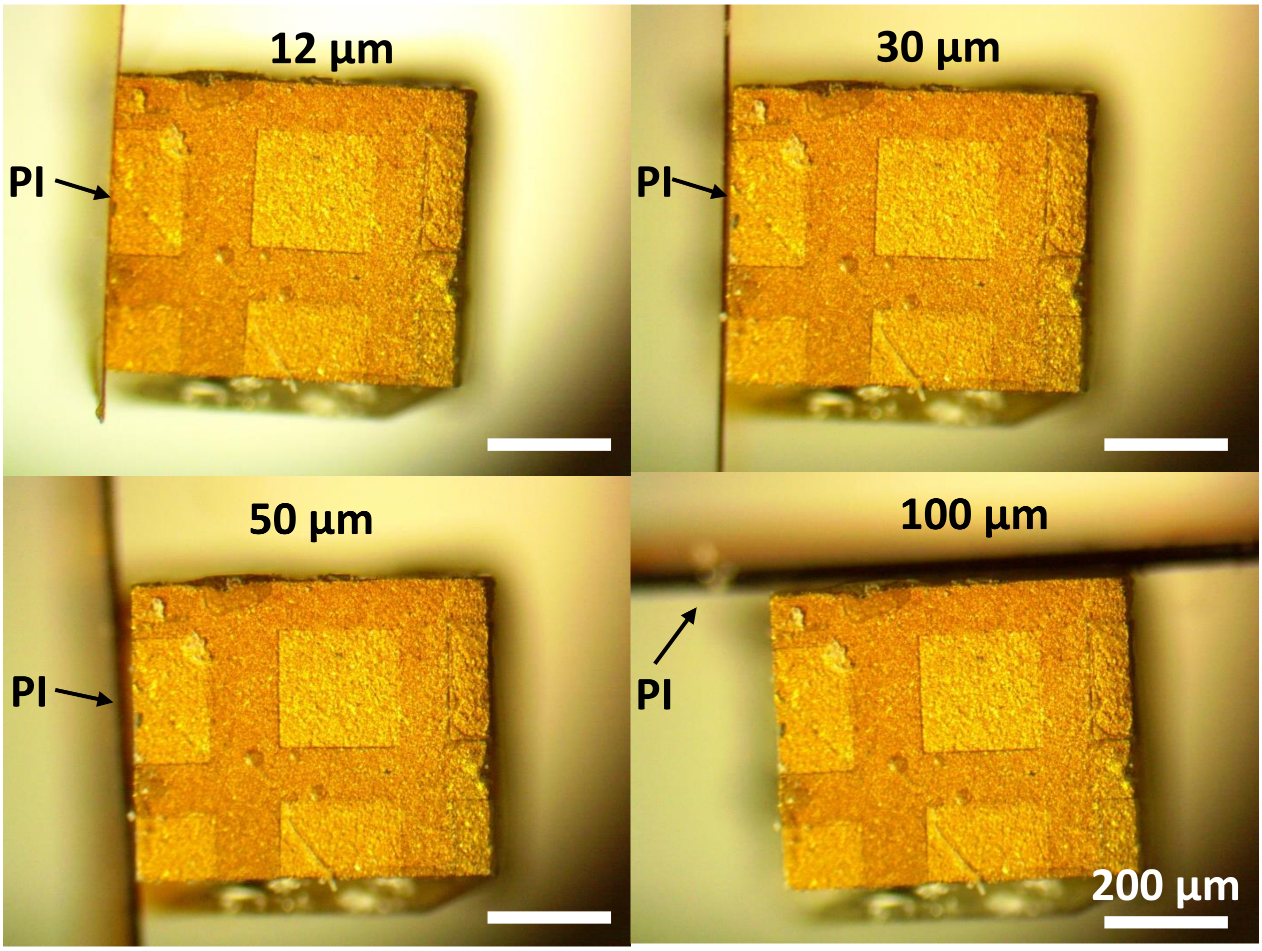

Fig S5: Optical images demonstrating the attachment of the PI film with various thicknesses on the PZT transducer element.

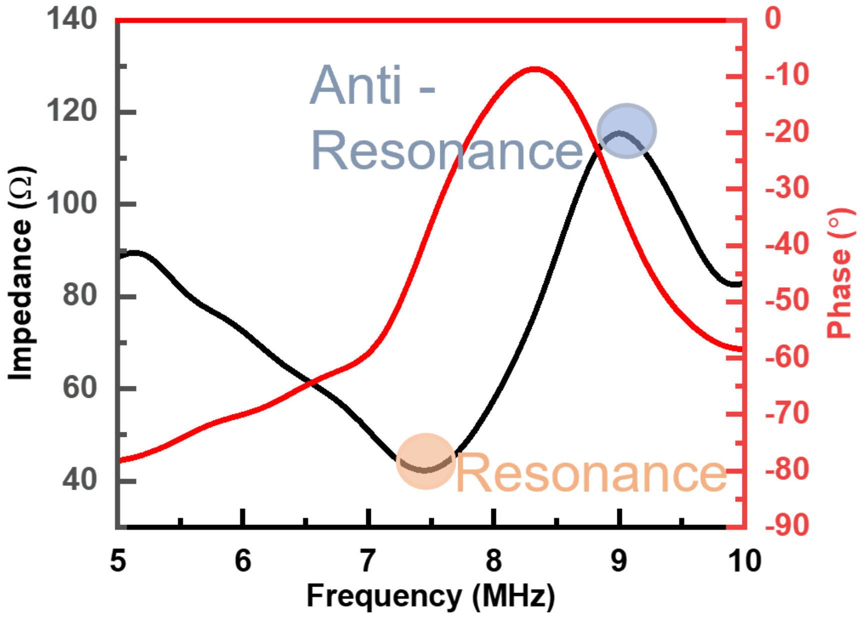

Fig S6: Impedance and phase angle spectrum of the 1-3 PZT composite with a thickness of 200 μm.

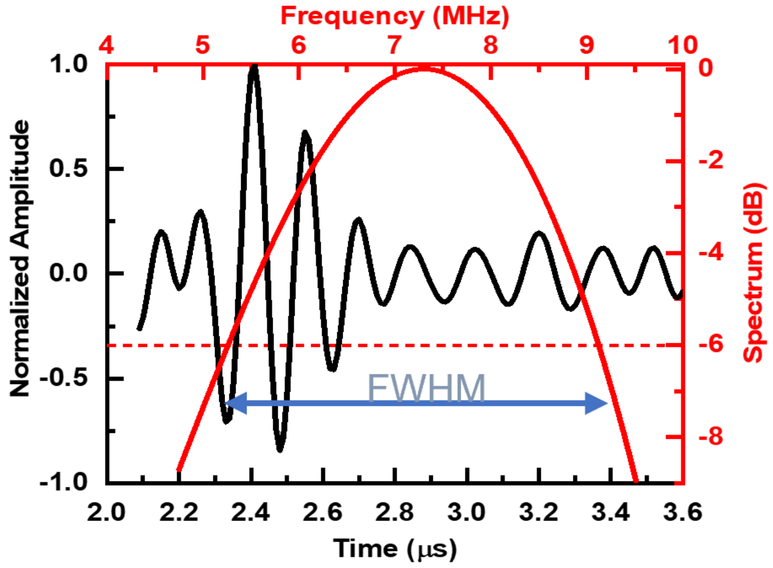

Fig S7: Time and frequency domain characterization of the 1-3 PZT composite with a thickness of 200 μm.

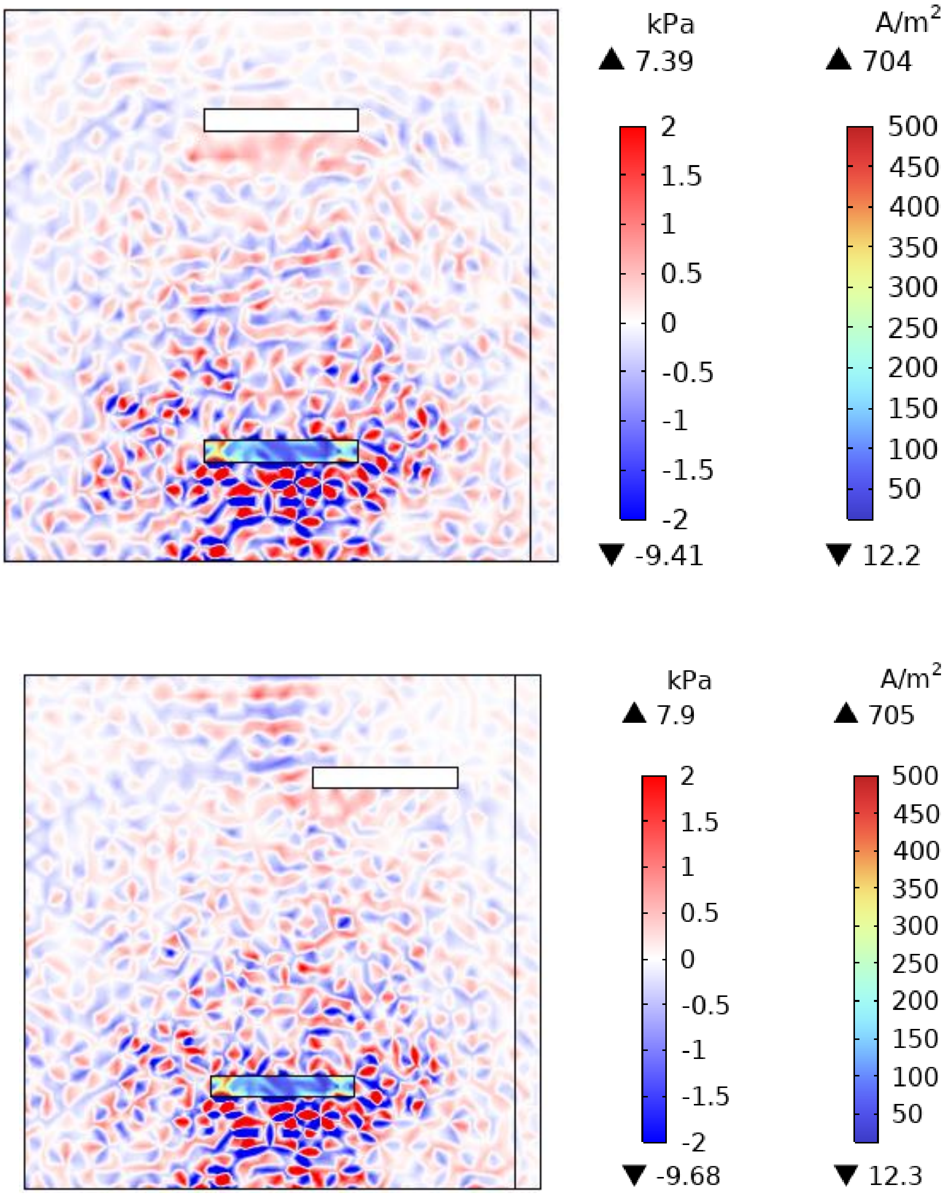

Fig S8: Invariance in the impedance or current density during the motion of the object from the middle (top) to the right (bottom) rules out the time-of-flight mechanism.

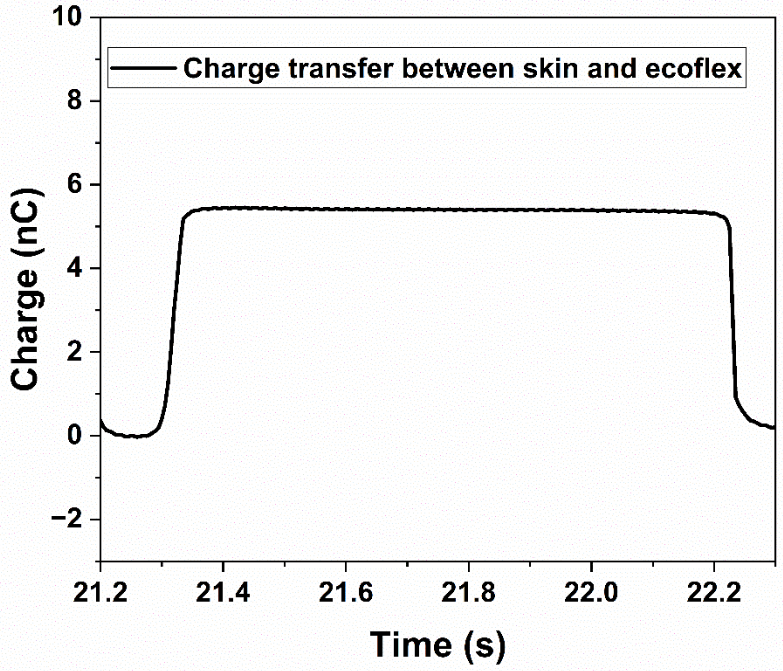

Fig S9: Triboelectric characterization between the skin and Ecoflex showing charge retention.

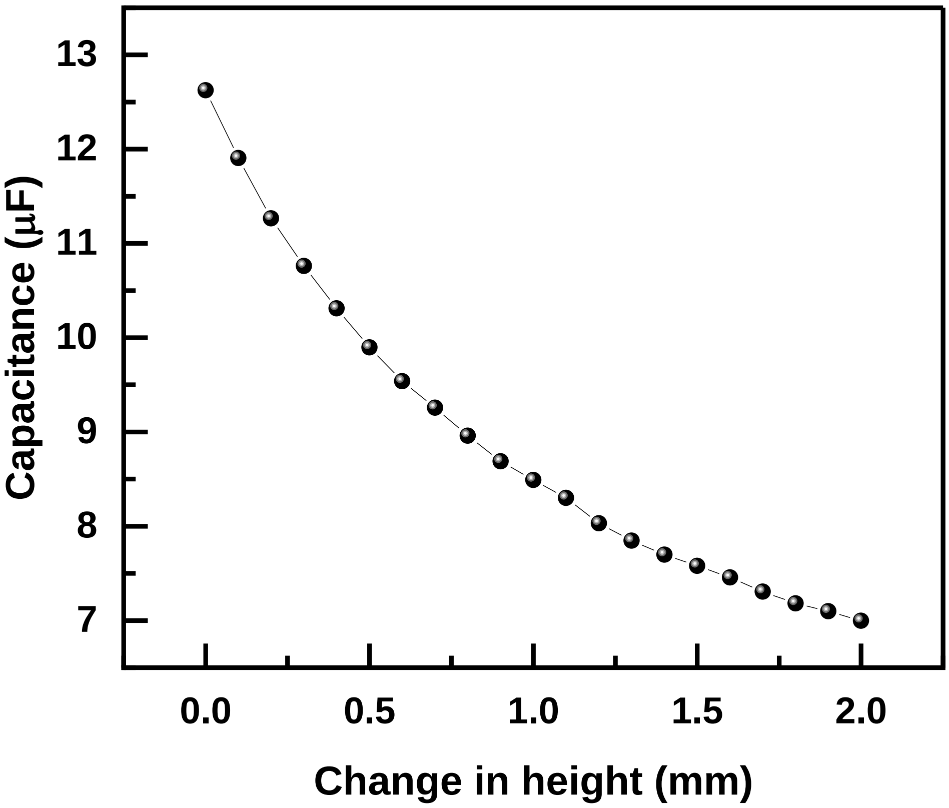

Fig S10: The simulated capacitance varies inversely proportional to the distance between the object and the ultrasound array.

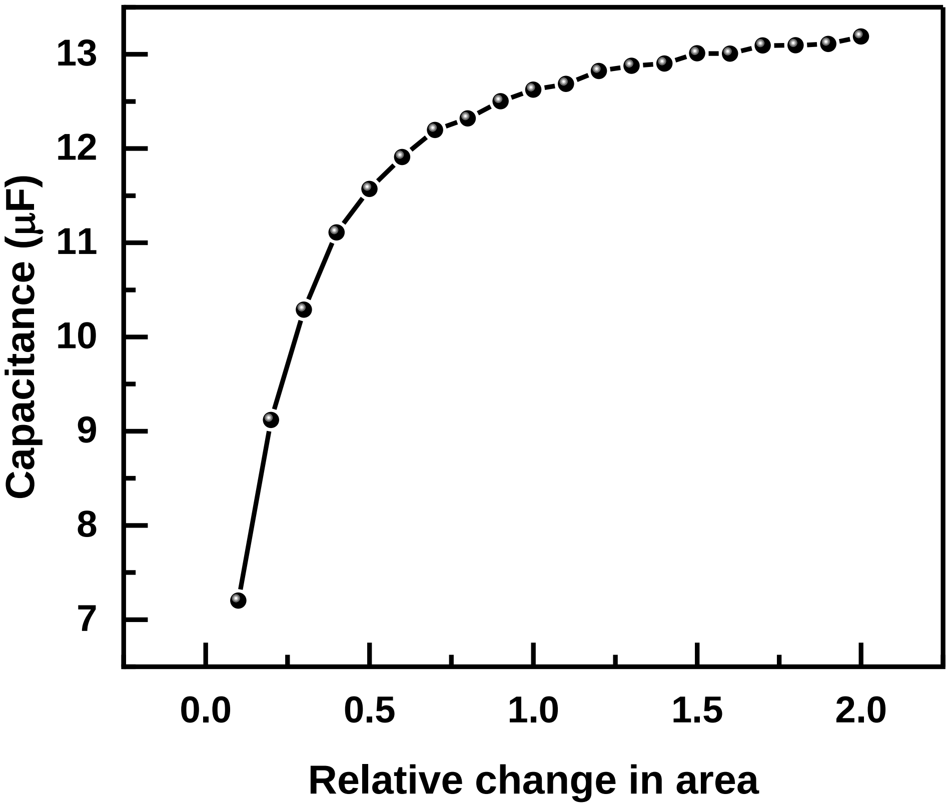

Fig S11: The simulated capacitance first rapidly increases and then saturates with the increasing area of the object.

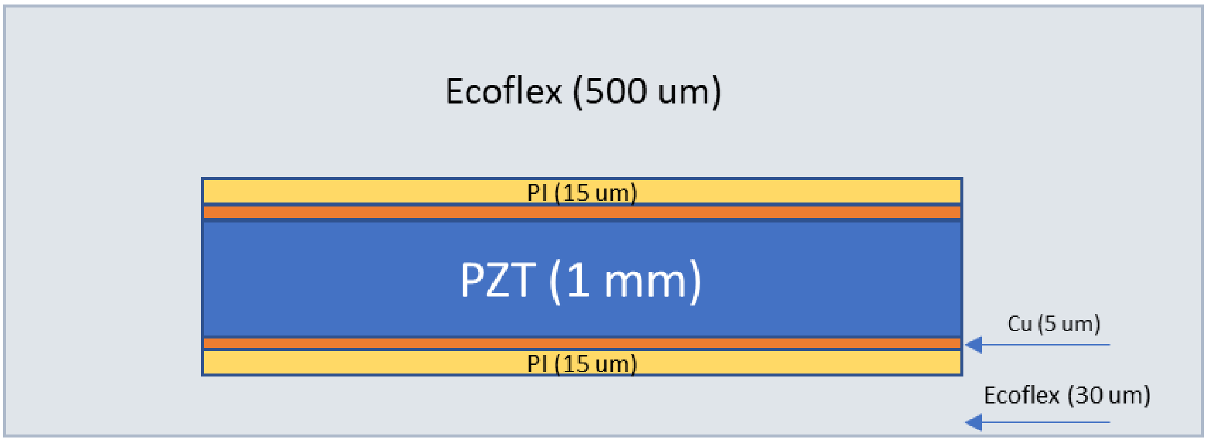

Fig S12: The cross-sectional view of each ultrasound element in the stretchable array.

Copper: $E = 90\ GPa,\ \upsilon = 0.34$

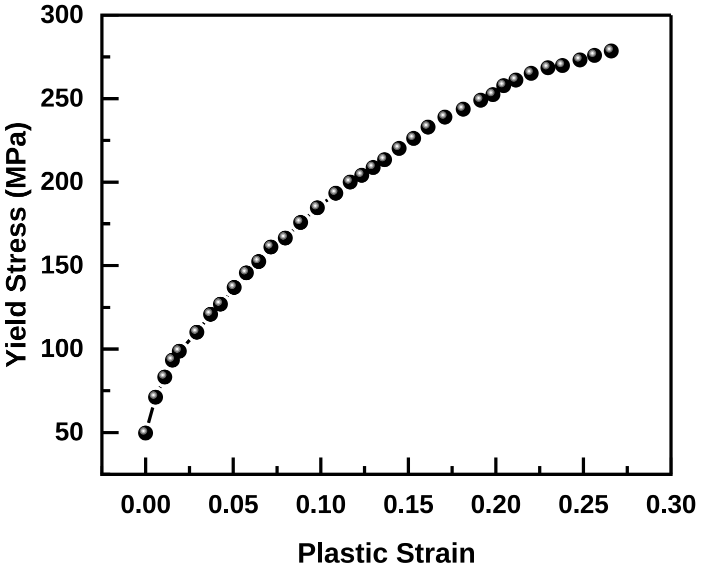

Polyimide: $E = 2.5\ GPa,\ \upsilon = 0.34$

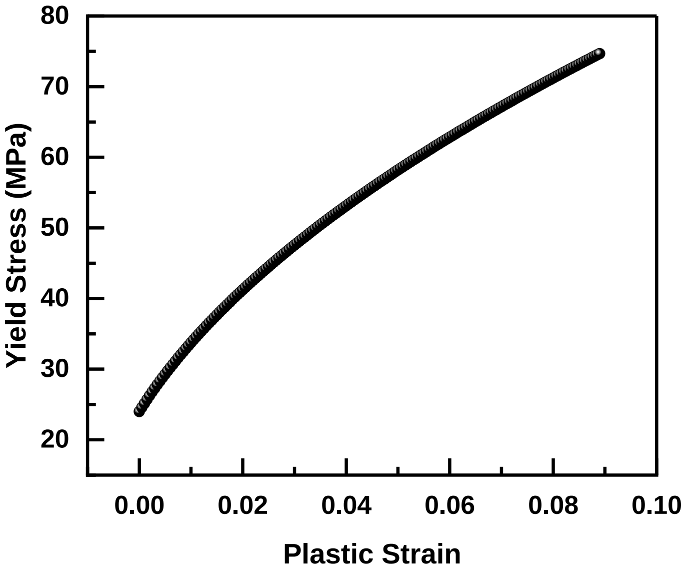

Fig S13: Yield stress and plastic strain of Cu/PI interconnects. The Young's modulus of Cu (or PI) is 90 GPa (or 2.5 GPa) and the Poisson ratio of Cu and PI is 0.34.

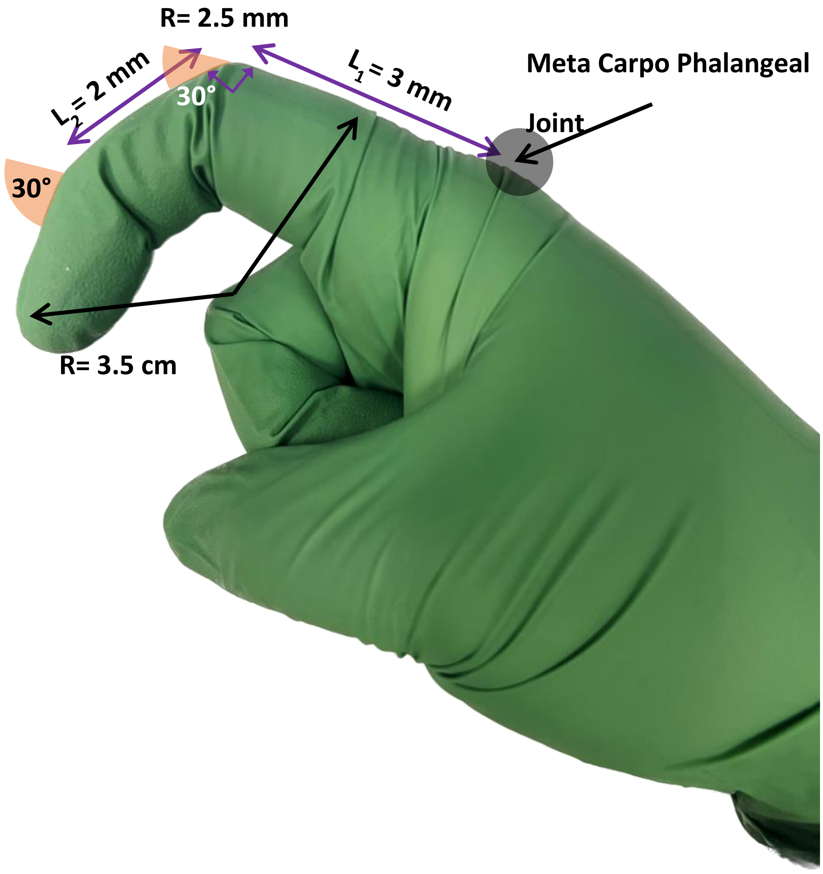

Fig S14: Invariance of triboelectricity during the change of the orientation or curvature (negative or positive) of the hand.

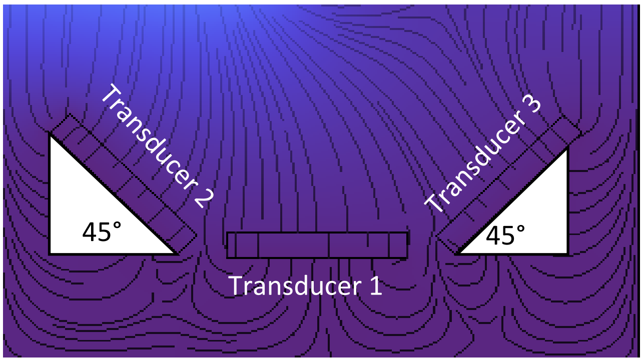

Fig S15: The orientation of different transducer elements in the array used for COMSOL simulation.

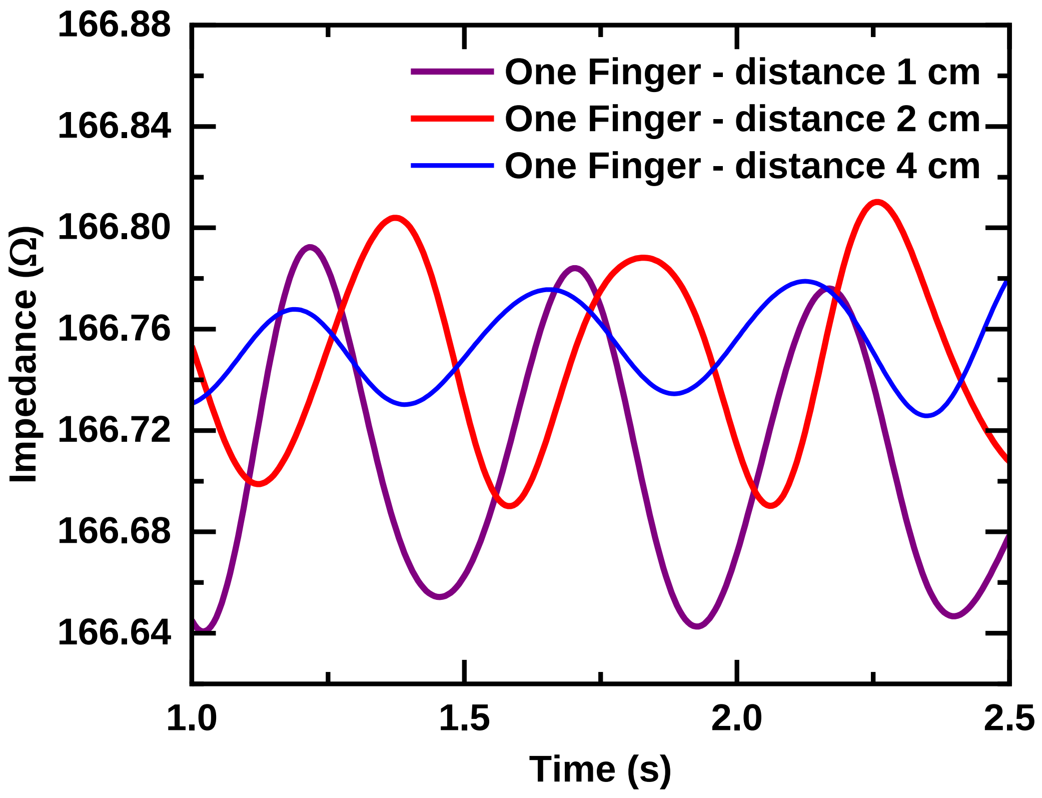

Fig S16: Temporal variation in the impedance during the vertical movement of one finger at varying distances: 1, 2, and 4 cm.

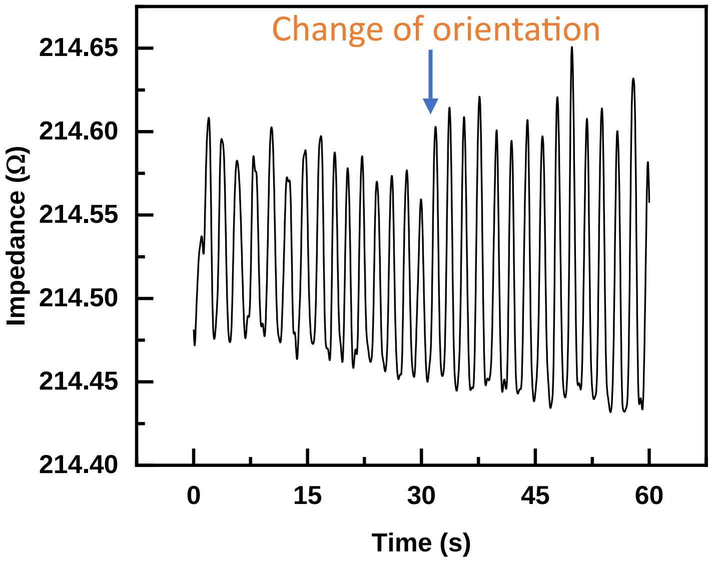

Fig S17: Invariance of triboelectricity during the change of the orientation or curvature (negative or positive) of the hand.

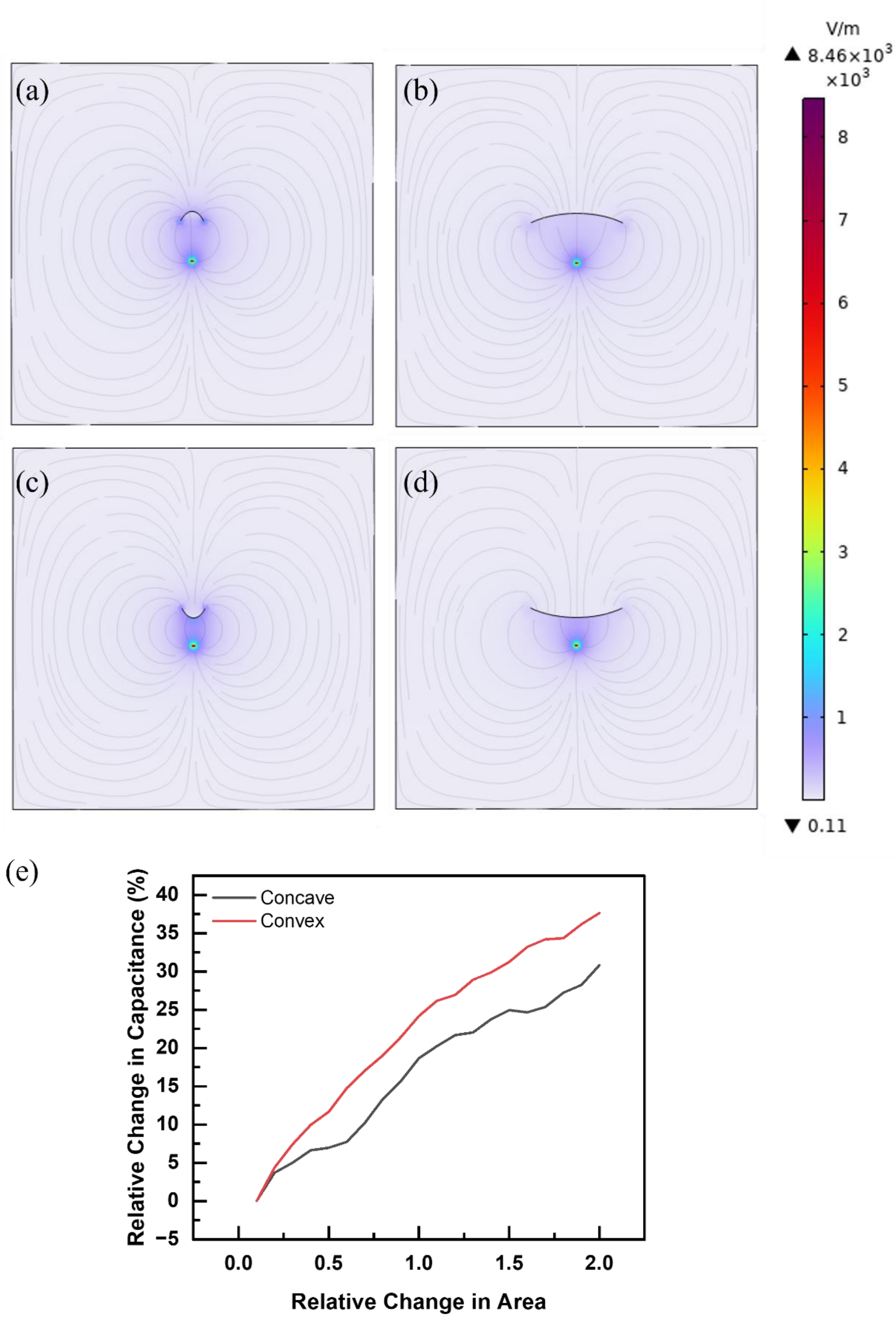

Fig S18: Variations of the time constant due to changes in orientation. Electric field distribution for **(a,b)** concave and **(c,d)** convex orientations with zoom-in shown in (**b,d**). **(e)** Relative change in capacitance as a function of the change in the geometric area for concave and convex orientations.

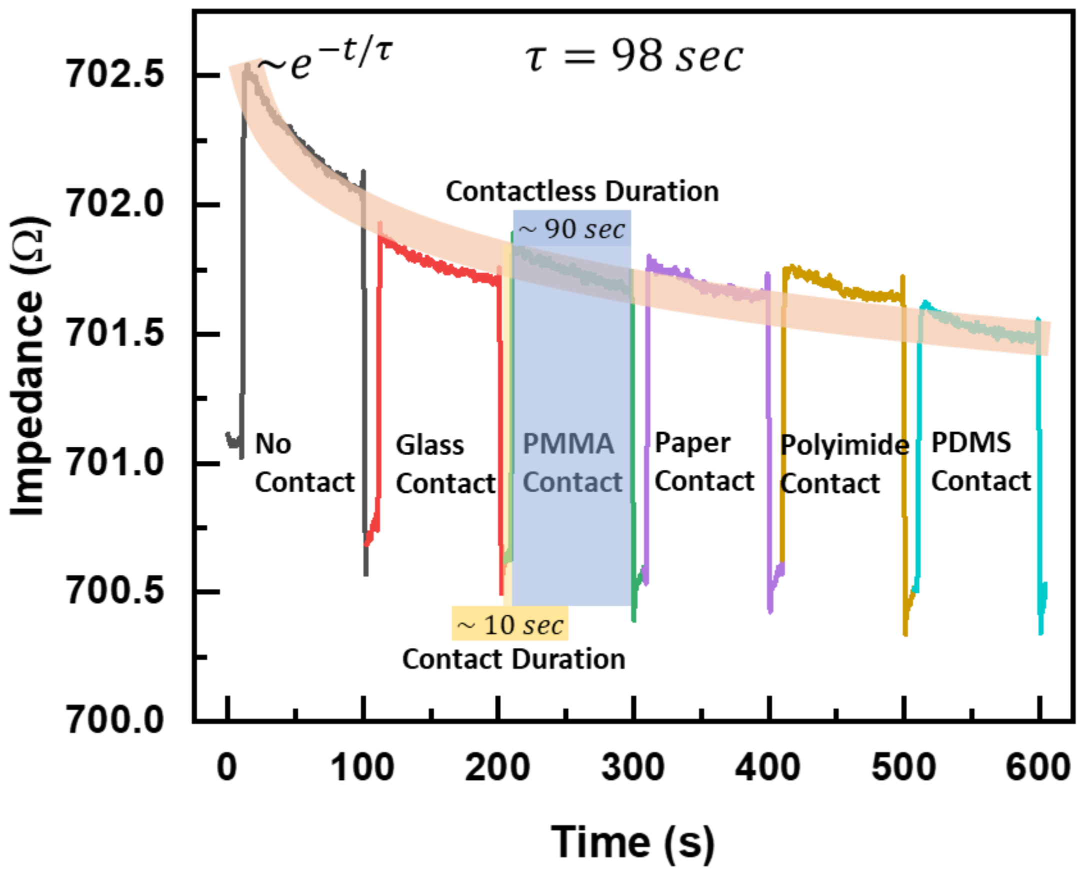

Fig S19: Invariance of the time decay constant during random surface contamination

References

[1] L. Chen, Q. Shi, Y. Sun, T. Nguyen, C. Lee and S. Soh, *Advanced Materials*, 2018, **30**, 1802405.

[2] C. Xu, B. Zhang, A. C. Wang, W. Cai, Y. Zi, P. Feng and Z. L. Wang, *Adv Funct Mater*, 2019, **29**, 1903142.
